# Presence of *IDH2* and *TP53* mutations significantly reduces survival of patients with chondrosarcoma

**DOI:** 10.1002/cncr.70376

**Published:** 2026-03-29

**Authors:** Anne Weidlich, Klaus‐Dieter Schaser, Tareq A. Juratli, Jessica Pablik, Sven Märdian, Kathrin Hauptmann, Philipp Schwabe, Stephan Richter, Hagen Fritzsche

**Affiliations:** ^1^ University Center for Orthopedics, Trauma Surgery, and Plastic Surgery Sarcoma Center National Center for Tumor Diseases German Cancer Research Center University Hospital Carl Gustav Carus Dresden Dresden Germany; ^2^ Department of Neurosurgery, Neurooncology Center National Center for Tumor Diseases German Cancer Research Center University Hospital Carl Gustav Carus Dresden Dresden Germany; ^3^ Institute of Pathology University Hospital Carl Gustav Carus Dresden Dresden Germany; ^4^ Center for Musculoskeletal Surgery Charité‐Berlin University Medicine Berlin Germany; ^5^ Institute of Pathology Charité‐Berlin University Medicine Berlin Germany; ^6^ Department for Trauma and Orthopedic Surgery, Center for Musculoskeletal Tumor Medicine Vivantes Hospital Spandau Berlin Germany; ^7^ Department of Medicine 1, Sarcoma Center National Center for Tumor Diseases German Cancer Research Center University Hospital Carl Gustav Carus Dresden Dresden Germany

**Keywords:** chondrosarcoma, IDH mutation, IDH2, sarcoma, survival, TP53

## Abstract

**Background:**

Chondrosarcoma (CS) has a prognosis largely influenced by tumor grade. Although *IDH* mutations have been reported in CS, impact on patient`s survival remains controversial. This study aims to assess prognostic relevance of *IDH* mutations on disease‐specific survival (DSS), metastasis‐free survival (MFS), and local recurrence‐free survival (RFS), in a large cohort of CS patients.

**Methods:**

The authors retrospectively analyzed CS samples from patients treated at two German musculoskeletal tumor centers. Tumor‐specific mutations, including *IDH*, *TP53*, *TERT promoter*, and *CDKN2A/B*, were identified through DNA isolation and next‐sequencing. Molecular findings were correlated with oncologic outcomes to evaluate their prognostic significance.

**Results:**

A total of 109 patients (53 females, 56 males; median age, 57 (16–89) years) were included. Tumor grades were G1/ACT (39%), G2 (41%), and G3/dedifferentiated (10%). Median follow‐up was 3.8 years with mortality of 17%. *IDH* mutations were detected in 59% (*IDH1*: 64%, *IDH2*: 36%), with dedifferentiated CS showing highest *IDH* mutation rate (91%), particularly *IDH2*. *IDH2*‐mutations were associated with significantly worse DSS compared to *IDH*–wild‐type or *IDH1*‐mutated tumors, independent of tumor grade. Specifically, *IDH1*‐*R132C* mutation correlated with significantly improved DSS compared to *R132G*, and *IDH2*‐*R172S* variant showed longest DSS and MFS, whereas *IDH2*‐*R172T* was linked to significant poor outcomes. In multivariable analysis, *IDH2* and *TP53* mutations were independent predictors of worse survival.

**Conclusion:**

*IDH2* and *TP53* mutations are enriched in dedifferentiated CS and are significant, independent predictors of adverse survival, regardless of tumor grade. These findings support *IDH2* mutation status as clinically meaningful prognostic biomarker that may allow risk stratification and clinical decision‐making in CS patients.

## Abstract 2

The presence of *IDH1* or *IDH2* mutations in chondrosarcomas varies depending on the grade of the tumor but their prognostic significance remains controversial. This study contributes to this evolving field by evaluating a sizably large cohort of chondrosarcoma patients and demonstrating that *IDH2* mutations are associated with significantly worse disease‐specific survival, which underscores the potential of *IDH* mutation subtypes as prognostic biomarkers in the clinical management of chondrosarcoma.

## INTRODUCTION

Chondrosarcomas (CS) are the second most common primary malignant bone tumors following osteosarcomas, accounting for approximately 26% of all bone tumors.^1^, ^2^ Among their subtypes, central conventional chondrosarcomas are the most frequent. These tumors are characterized by their comparatively poor response to radio‐ and chemotherapy, making wide, extralesional en bloc surgical resection the preferred and most effective treatment option.^3^, ^4^

In recent years, several molecular pathological findings relevant to prognosis have been identified. Mutations in *isocitrate dehydrogenase* (*IDH*) genes—originally found in gliomas—were first detected in central CS by Amary et al.^5^ *IDH1* and *IDH2* enzymes catalyze the reversible oxidative decarboxylation of isocitrate to alpha‐ketoglutarate (α‐KG) in the Krebs cycle. Mutant *IDH* enzymes instead produce 2‐hydroxyglutarate (2‐HG), an oncometabolite that accumulates in tumor cells and disrupts the function of α‐KG–dependent dioxygenases, including TET2 and histone demethylases, thereby altering epigenetic regulation.^6^, ^7^, ^8^, ^9^, ^10^ In addition, it has also been reported that an accumulation of the enantiomer D‐2‐HG probably contributes to increased malignant transformation and cancer.^11^, ^12^, ^13^, ^14^

*IDH* mutations are gain‐of‐function point mutations, most commonly *IDH1*‐*R132C* and *IDH2*‐*R172S* in chondrogenic tumors.^9^, ^15^, ^16^, ^17^ The frequency of *IDH* mutations depends on tumor grade—ranging from 50% in grade 2/3 to 87% in dedifferentiated CS—and is largely restricted to central CS and enchondromas.^2^ In contrast, other CS subtypes like clear cell, extraskeletal myxoid, and mesenchymal CS, as well as secondary peripheral CS, do not have *IDH* mutations and partly exhibit distinct genetic drivers (e.g., *EXT1/2* in peripheral CS). Additional recurrent alterations in CS, including *TP53*, *CDKN2A/B* deletions, and *TERT promoter* (*TERT‐p*) mutations—particularly enriched in dedifferentiated CS—allow to distinguish dedifferentiated from conventional CS and are linked to aggressive clinical behavior.^9^, ^18^, ^19^ Unlike gliomas, where *IDH* mutations are associated with a better prognosis, the prognostic relevance of *IDH* mutations in CS remains unresolved. Although some studies have suggested a correlation between *IDH*1/2 mutations and worse outcomes, others report no association or even improved progression‐free survival without impact on overall survival.^9^ The coexistence of *TERT‐p* mutations—more frequently observed in *IDH2*‐mutated tumors but apparently prognostically significant only in *IDH1*‐mutated tumors—further complicates interpretation of molecular findings.^19^ Moreover, epigenetic changes such as reversal of *IDH*‐induced hypermethylation have been associated with dedifferentiation, suggesting a complex interaction between molecular drivers and tumor progression.^18^, ^20^

This study aims to quantitatively assess the prognostic impact of *IDH* mutations, distinguishing between *IDH1* and *IDH2* variants, on disease‐specific (DSS), metastasis‐free (MFS), and local recurrence‐free survival (RFS), in a sizably large, well‐characterized cohort of CS patients from two university musculoskeletal tumor centers. Pursuing that approach, we perspectively seek to clarify the role of *IDH* mutations in clinical risk stratification and improve prognostic precision in chondrosarcoma management.

## MATERIALS AND METHODS

### Study design

In a retrospective multicenter study, all patients treated for CS at the University Center for Orthopaedics, Trauma and Plastic Surgery of the University Hospital Dresden (2002–May 2024) and at the Center for Musculoskeletal Surgery of the Charité, Berlin University Medicine (2010–May 2024) were included. After approval by the ethics committee (ethics vote no. BO‐EK‐135032023), clinical data (histology, molecular pathology, therapy, and follow‐up) were analyzed retrospectively.

Inclusion criteria were a histopathologically confirmed diagnosis of chondrosarcoma with subsequent tumor resection in a curative treatment setting. The exclusion criteria included histopathological evidence of mesenchymal CS, extraskeletal myxoid CS, clear cell CS, or secondary peripheral CS originating from cartilaginous exostosis, as these do not harbor *IDH* mutations as defined by the World Health Organization classification.^2^ Patients with a palliative therapy concept without tumor resection, patients with palliative surgery for tumor debulking, as well as those with missing information on *IDH* mutation status due to unavailable tumor tissue in the histopathological archive (*n* = 6) or nonvalid next‐generation sequencing (NGS) analysis due to unsuccessful or poor quality of DNA isolation (*n* = 16) were also excluded.

A total of 109 patients (Dresden, *n* = 86; Berlin, *n* = 23) who met the inclusion criteria with successful mutational analysis and complete clinical data were included in the final statistical analysis.

### DNA sequencing and mutation analysis

A detailed molecular pathological analysis of the existing mutation spectrum of the tumor was performed for all tumors using a clinically established NGS panel on biopsy tissue and/or specimens. For this purpose, the tumor areas were dissected from the formalin‐fixed, paraffin‐embedded material, and the DNA was extracted. The regions of interest (including *IDH*, *TERT‐p*, *TP53*, and *CDKNA/B*) were amplified using a custom‐designed amplicon panel according to the protocol “QIAseq Targeted DNA V3 Panel, May 2017” (QIAGEN, Hilden, Germany). In cases with unavailable matched normal tissue, tumor‐only sequencing was performed, as previously described.^21^, ^22^ The tumor‐only panel was custom‐designed by our group and manufactured by QIAGEN. The following targets were included: AKT1, ATRX, BRAF, CDKN2A, CIC, DAXX, EGFR, GNA11, GNAQ, H3F3A, H3F3B, IDH1, IDH2, KDM6A, KLF4, NF1, NF2, PIK3CA, PIK3R1, POLR2A, PTEN, SMO, STAG2, SUFU, TRAF7, TP53, and TERT. During library preparation, unique molecular barcodes and sample‐specific indices were incorporated according to the protocol. Indexed libraries were then paired‐end sequenced on the Illumina platform. HG19 (equivalent to GRCh37, release: February 2009) was used as the reference genome for bioinformatic analyses. The bioinformatics evaluation was performed using the CLC Biomedical Workbench (QIAGEN) using a customized analysis algorithm with the following filters: coverage ≥100, allele frequency ≥5%. Synonymous sequence variants and polymorphisms were not reported. Sequence variants with changes in amino acid sequence were reported according to HGVSc and HGVSp with respective allele frequencies.

### Statistics

The IBM SPSS Statistics program (version 28.0 for Windows) was used for the analyses (RRID: SPSS, RRID:SCR_002865). A heat map was established with R.^23^, ^24^ Our study examined male and female patients. Sex was initially tested as an independent variable in the univariate analysis using the log‐rank test. However, it did not reach statistical significance and was therefore not included in the multivariable Cox regression analysis. The social‐demographic and clinicopathological patient characteristics were analyzed descriptively by median, minimum, and maximum for nonnormal distributions, otherwise, variables were expressed as mean ± standard deviation. Tumor size and tumor volume were calculated based on pathology reports or preoperative magnetic resonance imaging (MRI) images. The recognized ABC2 formula for ellipsoid tumors was used for this calculation.^25^ Data were available from 77.1% (*n* = 84) of the patients. The remaining patients, for whom neither preoperative computed tomography nor MRI scans were found, nor the size was explicitly described in the histopathological report, have been excluded from the tumor volume calculations.

For measuring differences in variables such as age or tumor volume depending on the *IDH* mutation status, independent variables were first tested for normal distribution using the Shapiro‐Wilk test. For normal distribution, an analysis of variance homogeneity was performed using Levene’s test and, if this was confirmed, the ANOVA test—including the Games‐Howell test—was further used. If there was no normal distribution of the variables, the Kruskal–Wallis test was performed on more than two variables to test for differences between the groups.

Survival was calculated from the time of surgery to the last contact/date of death or first diagnosis of metastases or local recurrence. The occurrence of local recurrences, metastases, and deaths was analyzed to calculate the DSS, MFS, and RFS of the patients. Survival was estimated according to the Kaplan–Meier method and compared using the log‐rank test. Cox regression analysis was performed to identify possible prognostic factors influencing survival time. In this study, unless otherwise specified, the significance level was 5% on both sides, and a 95% CI was presented.

## RESULTS

### Clinical characteristics

**FIGURE 1:**
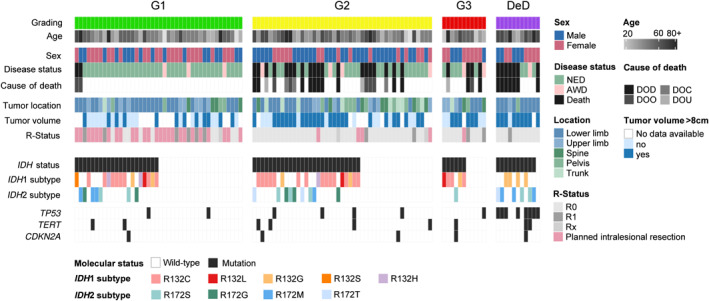
Heatmap with individual tumor features/patients characteristics.

Of 109 patients included, 51.4% (*n* = 56) were male and 48.6% (*n* = 53) female. Histopathologically, a G1‐CS or atypical cartilaginous tumor (ACT) was found in 38.5% (*n* = 42), G2 in 41.3% (*n* = 45), and G3 and dedifferentiated CS in 10.1% (*n* = 11), respectively (Figure 1 and Table 1, respectively). The median age at the time of surgery was 57 (16–89) years. Moreover, we noticed a high rate of patients beyond 60 years in the group of dedifferentiated CS (72.7%, *n* = 8).

**TABLE 1:** Clinical characteristics.

	All, *n* = 109 (%)	*IDH* wild‐type, *n* = 45 (41.3%)	*IDH1* mutation, *n* = 41 (37.6%)	*IDH2* mutation, *n* = 23 (21.1%)	χ^2^ test, *p*
Sex					.695
Female	53 (48.6)	24 (53.3)	19 (46.3)	10 (43.5)	
Male	56 (51.4)	21 (46.7)	22 (53.7)	13 (56.5)	
Age at time of surgery (years) (mean ± SD)	56.8 (±16.5)	51.3 (±17.3)	58.0 (±15.6)	65.6 (±12.3)	.002^a^ (ANOVA)
Age at time of surgery >60 years	47 (43.1)	13 (28.9)	20 (48.8)	14 (60.9)	.008^a^
Grading					
G1/ACT	42 (38.5)	21 (46.7)	14 (34.1)	7 (30.4)	.328
G2	45 (41.3)	18 (40.0)	18 (43.9)	9 (39.1)	.934
G3	11 (10.1)	5 (11.1)	5 (12.2)	1 (4.3)	.580
Dedifferentiated	11 (10.1)	1 (2.2)	4 (9.8)	6 (26.1)	.008^a^
Localization					
Upper extremities	20 (18.3)	8 (17.8)	8 (19.5)	4 (17.4)	<.001^a^
Lower extremities	52 (47.7)	15 (33.3)	18 (43.9)	19 (82.6)	<.001^a^
Pelvis	14 (12.8)	8 (17.8)	6 (14.6)	—	.106
Spine	13 (11.9)	10 (22.2)	3 (7.3)	—	.014^a^
Chest wall/scapula	10 (9.2)	4 (8.9)	6 (14.6)	—	.150
Tumor volume (cm^3^), (median range)^b^	103.7 (2.3–5544)	98.0 (2.4–5544)	77.8 (2.3–1212)	150.0 (2.5–2095)	.744 (Kruskal–Wallis)
Tumor size >8 cm^b^	52/84 (61.9)	18/31 (58.1)	18/32 (56.3)	16/21 (76.2)	.294
Precursor lesion	5 (4.6)	1 (2.2)	4 (9.8)	—	.123
Resection status/surgical margins					.190
R0	64 (58.7)	21 (46.7)	27 (65.9)	16 (69.6)	
R1	11 (10.1)	8 (17.8)	2 (4.9)	1 (4.3)	
Rx	3 (2.8)	2 (4.4)	—	1 (4.3)	
Planned intralesional/R2	31 (28.4)	14 (31.1)	12 (29.3)	5 (21.7)	
Multimodal therapy					
Neoadjuvant chemotherapy	12 (11.0)	5 (11.1)	6 (14.6)	1 (4.3)	.451
Neoadjuvant radiation	2 (1.8)	2 (4.4)	—	—	.235
Adjuvant chemotherapy	13 (11.9)	6 (13.3)	4 (9.8)	3 (13.0)	.862
Adjuvant radiation	13 (11.9)	5 (11.1)	5 (12.2)	3 (13.0)	.971

^a^ Significant in 95% confidence interval.

^b^ *n* = 84 patients.

CS was localized in the lower and upper extremities in 47.7% (*n* = 52) and 18.3% (*n* = 20), respectively. Other sites were the pelvis 12.8% (*n* = 14), the spine in 11.9% (*n* = 13), and the chest wall/scapula in 9.2% (*n* = 10).

Data on tumor volume or tumor size were available for 77.1% (*n* = 84) of patients, with a median of 103.7 cm^3^ (2.3–5544.0), and 52 patients (61.9%) had tumors measuring at least 8 cm in size. A precursor lesion, such as Ollier’s disease, was present in 4.6% of patients (*n* = 5).

Surgical treatment of ACTs was performed by excochleation and palacoplasty or combined with osteosynthesis. The aim for all CS patients was a complete tumor excision, either gross‐total (selected patients with G1‐CS sarcoma) or extralesional en bloc (all patients with G2/3‐CS). Tumor‐free surgical margins (R0) were achieved in 81% of all patients, indicating an R0‐ resection rate for all G2, G3, and dedifferentiated CS of 90.2%, 80%, and 90.9%, respectively. Resulting bone defects close to joints were reconstructed using endoprosthetic replacement, in diaphyseal long bone segments using either biological reconstructions (free non‐/vascularized autologous bone transfer/segment transport) or modular replacement systems. Reconstruction of spinal defects (following en bloc spondylectomy) was accomplished with posterior transpedicular screw/rod fixation and anterior vertebral body replacement systems/cages.

Multimodal therapy was, according to the currently valid guideline, only applied to patients with dedifferentiated CS. Six patients with G3‐CS also received neoadjuvant or adjuvant chemotherapy, as an individual therapeutic decision at the tumor conference. Eleven percent of patients received neoadjuvant systemic therapy, whereas 12% underwent adjuvant systemic therapy. In eight patients, high‐grade or dedifferentiated CS was only detected in the final specimen, as no biopsy was performed (*n* = 2) or a lower grade was detected in the biopsy without an indication for neoadjuvant therapy (*n* = 6). Radiotherapy was generally given as an adjuvant treatment (12%) and only 2% in a neoadjuvant setting. Three patients received adjuvant combined radio‐chemotherapy.

### Mutation analysis

**FIGURE 2:**
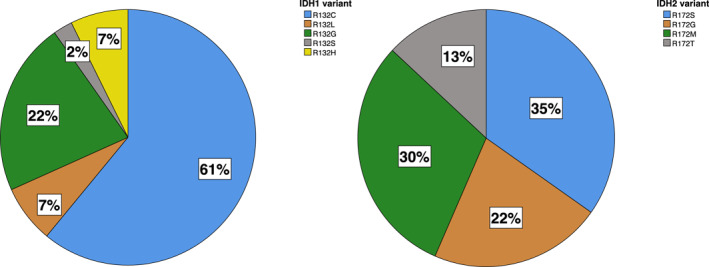
Percentage of *IDH1* and *IDH2* variants.

An *IDH* mutation was detected in 58.7% of patients (*n* = 64). At 37.6%, the *IDH1* mutation (*n* = 41) was more common than the *IDH2* mutation (*n* = 23, 21.1%). The *IDH1‐R132C* and the *IDH2‐R172S* mutations were detected most frequently (61% of all *IDH1* mutants and 34.8% of all *IDH2* mutants, respectively) (Figure 2).

Remarkably, patients with *IDH2*‐mutant CS were significantly older (65.6 years; 95% CI, 60.3–70.9) than their counterparts with *IDH‐WT* (51.3 years; 95% CI, 46.1–56.5) or *IDH1*‐mutated CS (58.0 years; 95% CI, 53.0–62.9, *p* = .002). Dedifferentiated CS were enriched with *IDH* mutations (10 of 11 patients; 91%), in particular, *IDH2* mutations were significantly more frequent in dedifferentiated CS (26%, *p* = .008).

*IDH2*‐mutated CS were only localized in the extremities, especially in the lower extremities (*n* = 19, 82.6%). In CS of the chest wall/scapula, the spine (*p* = .014), and the pelvis, only *IDH‐WT* or *IDH1* mutation could be identified.

The tumor volume was not normally distributed for *IDH‐WT*‐CS, *IDH1*‐mutated, and *IDH2*‐mutated CS (Shapiro‐Wilk test, *p* < .001) without significant differences in tumor volume between *IDH1*‐mutated, *IDH2*‐mutated, and *IDH‐WT*‐CS patients (Kruskal–Wallis test; *p* = .744). Additionally, an analysis of tumors that were equal to or larger than 8 cm in at least one dimension was performed. Patients with CS larger than 8 cm tended to have a higher proportion of *IDH2*‐mutations (76.2%) when compared to *IDH1*‐mutated (56.3%) and *IDH‐WT* CS (58.1%).

In addition, further mutations were analyzed (Table S1), including *TP53* mutations in 13.8% (*n* = 15) with 8.9% in *IDH‐WT* (*n* = 4), 17.1% in *IDH1*‐mutated (*n* = 7), and 13.8% in *IDH2*‐mutated (*n* = 4) CS without any significant differences. However, the *TP53* mutation in dedifferentiated CS with 72.7% (*n* = 8) was significantly more frequent than in G1‐CS (4.8%, *n* = 2), G2‐CS (8.9%, *n* = 4), and G3‐CS (9.1%, *n* = 1), *p* < .001.

*TERT‐p* mutations were found in 8.3% of tumors (*n* = 9). They were with 2.2% lowest in IDH‐WT (*n* = 1) and enriched in IDH1‐mutated (12.2%, *n* = 5) and IDH2‐mutated (13%, *n* = 3) CS. *CDKN2A/B* deletions were only detected in 4.6% (*n* = 5) of patients with 2.2% in *IDH‐WT* (*n* = 1), 4.9% in *IDH1*‐mutated (*n* = 2), and highest in *IDH2*‐mutated CS (8.7%, *n* = 2). There was no co‐accuracy between *TP53* mutation (*p* = .464), *TERT‐p* mutation (*p* = .157), as well as *CDKN2A/B* deletion (*p* = .480) and the *IDH* mutation status.

### Survival analysis

The median follow‐up (FU) was 3.8 years (1–238 months). Overall, 30.3% (*n* = 33) of patients died, in total, 17.4% were tumor‐related (Table 2). Estimated DSS according to Kaplan–Meier was 15.7 years (95% CI, 14.0–17.4) with significant worse DSS of patients with *IDH2*‐mutated CS (*IDH‐WT*: 210 months; 95% CI, 187.0–232.8; *IDH1*: 163 months; 95% CI, 137.2–189.5; *IDH2*: 97 months; 95% CI, 54.6–139.3). This results in a 1‐, 2‐, 5‐, and 10‐year survival rate of 93%, 87%, 81%, and 79%, respectively. A local recurrence was seen in 23% after a median of 10 (1–151) months, in 36% a histopathological higher grading of the recurrent lesion was seen and in 32% more than one local recurrence was detected during the course of the disease.

**TABLE 2:** Outcome data.

	All, *n* = 109 (%)	*IDH* wild‐type, *n* = 45 (41.3%)	*IDH1* mutation, *n* = 41 (37.6%)	*IDH2* mutation, *n* = 23 (21.1%)	χ^2^ test, *p*
Status of last follow‐up					
NED	66 (60.6)	29 (64.4)	27 (65.9)	10 (43.5)	.167
AWD	10 (9.2)	5 (11.1)	4 (9.8)	1 (4.3)	.650
DOD	19 (17.4)	5 (11.1)	6 (14.6)	8 (34.8)	.043^a^
DOO	8 (7.3)	4 (8.9)	2 (4.9)	2 (8.7)	.746
DOC	3 (2.8)	1 (2.2)	‐	2 (8.7)	.120
DOU	3 (2.8)	1 (2.2)	2 (4.9)	‐	.499
Distant metastasis^b^	28 (26.2)	10 (22.7)	11 (27.5)	7 (30.4)	.770
Local recurrence	25 (22.9)	12 (26.7)	8 (19.5)	5 (21.7)	.724
Upgrading of recurrence	9 (36.0)	4 (33.3)	4 (50.0)	1 (20.0)	.529
Multiple recurrences	8 (32.0)	5 (41.7)	3 (37.5)	‐	.225

Abbreviations: AWD, alive with disease; DOC, dead of complications; DOD, dead of disease; DOO, dead of other causes; DOU, dead of unknown causes; NED, no evidence of disease.

^a^ Significant in 95% confidence interval.

^b^ *n* = 107 (without two patients with primary metastases).

Thus, resulting in a Kaplan–Meier estimated RFS of 167 months (95% CI, 143.2–190.9) with worst survival of patients with *IDH2*‐mutated CS (*IDH‐WT*: 164 months; 95% CI, 128.5–199.6; *IDH1*: 149 months; 95% CI, 118.6–180.1; *IDH2*: 124 months; 95% CI, 83.5–164.3). Twenty‐six percent developed secondary metastases after a median of 9 (1–156) months, predominantly pulmonary, but in some patients also lymphogenic, osseous, dermal, in soft tissue or peritoneal metastases. According to Kaplan–Meier, the estimated MFS was 166 months (95% CI, 143.9–188.8) with the worst outcome of patients with *IDH2*‐mutated CS (110 months; 95% CI, 70.3–150.2), compared to *IDH‐WT* (182 months; 95% CI, 151.8–211.9) and *IDH1*‐mutated patients with 136 months (95% CI, 106.4–166.1).

Primary pulmonary metastases were found in two patients, which should be resected subsequently in an individual curative treatment setting. These two patients were therefore excluded, resulting in a total number of 107 patients who were used for analysis of MFS, whereas the evaluation of DSS and RFS was performed for all 109 patients.

In a univariate regression analysis using the log‐rank test, factors influencing survival were analyzed and DSS, RFS, and MFS were estimated according to Kaplan–Meier (Table S2).

Unsurprisingly, the histopathological grading significantly influenced DSS, RFS, and MFS (*p* < .001 each): higher tumor grades were associated with progressively poorer survival outcomes, with the worst survival observed in patients with dedifferentiated CS (Figure S2).

Age over 60 years had a significant negative effect on DSS (*p* = .008) of CS patients in our study. Tumor size also seemed to represent an influencing factor on survival: Starting from a tumor dimension of at least 8 cm and upward, the patients had a significantly worse DSS (*p* = .007), MFS (*p* = .039), and RFS (*p* < .001).

Patients with *IDH2* mutation had significantly worse DSS (estimated according to Kaplan–Meier: 97 months; 95% CI, 55–139) regardless of the histopathological grading (*p* = .001) (Figure 1). In contrast, no significant differences in survival were observed between patients with *IDH‐WT* CS and those with *IDH1*‐mutated CS, with Kaplan–Meier estimates of 210 months (95% CI, 187–233) and 163 months (95% CI, 137–190), respectively. A significant variation in DSS was noted depending on the specific subtype of *IDH1* and *IDH2* mutations. Among *IDH1* mutations, patients with *R132C* mutation demonstrated a significantly better survival compared to those with the *R132G* mutation (*p* = .007) but worse survival relative to patients with the *R132H* mutation (*p* = .034). For *IDH2* mutations, patients with the *R172S* variant had the longest survival, showing a significant advantage over those with the *R172T* mutation (*p* < .001), which was associated with the shortest survival. Additionally, patients with the *R172M* mutation had significantly better survival than those with the *R172T* mutation (*p* = .007). Furthermore, the age of the patients appeared to have a significant impact on survival outcomes. Specifically, patients over 60 years old at the time of surgery showed significantly worse DSS (*p* = .018).

**FIGURE 3:**
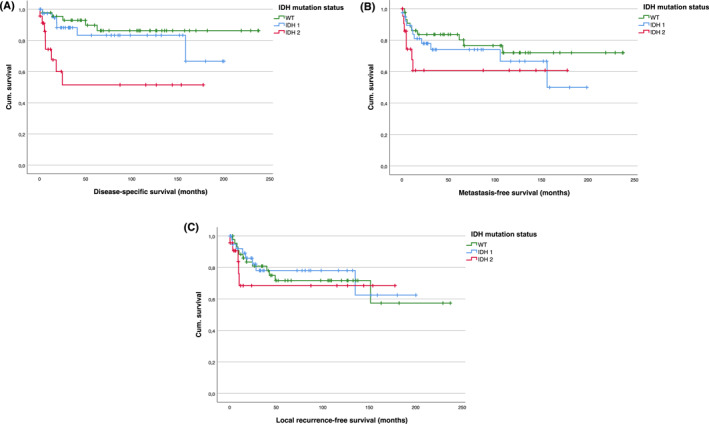
(A) DSS, (B) MFS, and (C) RFS regarding *IDH*‐mutation, differentiated into *IDH1* and *IDH2*‐mutation. (A) Significant differences in DSS (*p* = .001): *IDH‐WT* versus *IDH1/IDH2* (*p* = .388/<.001, respectively); *IDH1* versus *IDH2* (*p* = .009). (B) No significant differences in MFS: *IDH‐WT* versus *IDH1/IDH2* (*p* = .389/.131, respectively); *IDH1* versus *IDH2* (*p* = .322). (C): No significant differences in RFS: *IDH‐WT* versus *IDH1/IDH2* (*p* = .795/.534, respectively); *IDH1* versus *IDH2* (*p* = .407).

Regarding RFS, no influence of the *IDH* mutation status (Figure 3) or resection status was observed, whereas there was a significantly worse RFS in patients with adjuvant chemotherapy (Table S2).

For MFS, estimated by Kaplan–Meier analysis at 166 months (95% CI, 144–189), only a trend toward worse survival was observed in patients with *IDH2*‐mutated CS (110 months; 95% CI, 70–150) compared to those with *IDH‐WT* CS (182 months; 95% CI, 152–212) and *IDH1*‐mutated CS (137 months; 95% CI, 106–166) (Figure 3). A significant difference in MFS was again noted based on the *IDH2* mutation subtype. Patients with *R172S* and *R172M* mutations demonstrated significantly better survival compared to those with the *R172T* mutation (*p* < .001 and *p* = .002, respectively), the last being associated with the shortest survival time.

Furthermore, *TP53* mutation was also associated with significantly worse DSS, RFS, and MFS (*p* < .001 each) (Figure S1). A tumor size larger than 8 cm was another significant negative influencing factor for the DSS (*p* = .007), RFS (*p* < .001), and MFS (*p* = .039).

**FIGURE 4:**
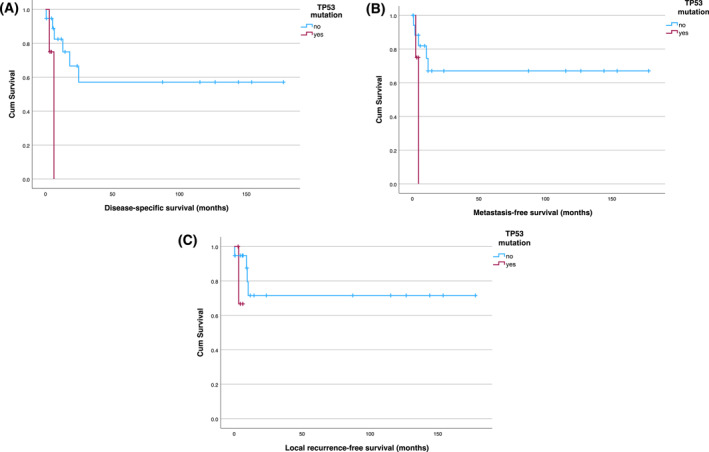
(A) DSS, (B) MFS, and (C) RFS in patients with *IDH2* mutation regarding *TP53*‐mutation. (A) Significant differences in DSS (*p* = .019). (B) No significant differences in MFS (*p* = .101). (C) No significant differences in RFS (*p* = .204).

Among the 23 *IDH2*‐mutated patients, four patients (17.4%) had a *TP53* co‐mutation. However, only patients with dedifferentiated CS (66.7%) were found to have both mutations, whereas in patients with conventional CS, the combination of *IDH2* and *TP53* mutations were not detectable. In *IDH2*‐mutated patients, a *TP53* co‐mutation had a significantly negative influence (*p* = .019) in respect to DSS, whereas no significant differences were observed regarding MFS and RFS (*p* = .101 and *p* = .204, respectively) (Figure 4).

In Cox regression analysis, the clinically relevant variables that were significant in the univariate analysis (log‐rank test) were included (Table 3). Although no significant influence on DSS could be shown for the patients’ age in the Cox regression analysis, there was a significant negative prognostic influence for patients with dedifferentiated CS and the *IDH2* mutation. The variables did not differ in their extent of influence.

**TABLE 3:** Cox regression analysis for DSS, MFS, and RFS.

	Univariate (log‐rank), *p*	Multivariate (Cox model), *p*	Relative risk (95% CI)
Disease‐specific survival			
Age >60 years	.018^a^	.144	—
G3‐CS	<.001^a^	.361	—
Dedifferentiated CS	<.001^a^	.005^a^	8.94 (1.93–41.56)
*IDH2* mutation	.001^a^	.020^a^	3.61 (1.22–10.67)
*TP53* mutation	<.001^a^	.127	—
Metastasis‐free survival			
Low‐grade CS (G1‐CS)	<.001^a^	.002^a^	0.93 (0.02–0.41)
G3‐CS	<.001^a^	.678	—
Dedifferentiated CS	<.001^a^	.710	—
*IDH2* mutation	.255	.088	—
*TP53* mutation	<.001^a^	<.001^a^	12.82 (4.57–35.94)
Local recurrence‐free survival			
Low‐grade CS (G1‐CS)	<.001^a^	.045^a^	0.37 (0.14–0.98)
G3‐CS	<.001^a^	.314	—
Dedifferentiated CS	<.001^a^	.323	—
*IDH2* mutation	.702	.623	—
*TP53* mutation	<.001^a^	.046^a^	3.22 (1.02–10.17)

Abbreviation: CS, chondrosarcoma.

^a^ Significant in 95% CI.

Regarding MFS and RFS, the *IDH2* mutation was not a significant influencing factor in the Cox regression analysis according to the nonsignificant results in the log‐rank test, whereas the *TP53* mutation had a negative impact on MFS and RFS. In contrast, a low‐grade histopathology was a significantly predictive factor for a better MFS and RFS.

An additional Cox regression analysis with 84 patients for which the tumor size was available revealed no significant influence of a tumor size larger than 8 cm on DSS, MFS, or RFS.

## DISCUSSION

Among the newly emerging prognostic factors known to influence the course and progression of CS disease, the well documented cancer‐associated mutation of *IDH* has attracted major interest. Because Amary et al.^5^ succeeded in detecting the *IDH* mutation in CS, the prognostic impact of *IDH* mutations in CS remains controversial, and conclusive evidence on prognostic value is missing. In this multicenter study, we investigated the prognostic relevance of *IDH1* and *IDH2* mutations in CS using a well‐characterized cohort of 109 patients from two major sarcoma centers. To our knowledge, our analysis is the first to show that *IDH2* mutations are specifically enriched in dedifferentiated CS and are significantly associated with older age, extremity localization, and worse DSS, regardless of tumor grading. These findings shed new light on the distinct clinical behavior and molecular landscape of *IDH2*‐mutated CS and propose *IDH2* as a clinically relevant biomarker for prognosis.

Our study confirms the overall frequency of *IDH* mutations in CS at 59%, with *IDH1* and *IDH2* mutations detected in 38% and 21% of patients, respectively. These frequencies are consistent with previously reported ranges of 34%–74% in the literature.^9^, ^17^, ^26^, ^27^, ^28^, ^29^ Among the variants, *IDH1*‐*R132C* (60%) and *IDH2*‐*R172S* (35%) were the most common, aligning with prior reports.^9^, ^15^, ^16^, ^17^ However, our data go further by providing a detailed correlation between specific *IDH2* variants and clinical outcomes, highlighting the negative prognostic impact of the *IDH2*‐*R172T* mutation. Future studies with larger patient numbers than those of Trovarelli et al.^28^ and ours are needed to reveal if this finding can be used for risk stratification of patients. Identification of a subset of *IDH1‐R132G*‐mutated or *IDH2‐R172T*‐mutated patients with comparatively higher risk may have implications for a very early therapeutic adjustment. Pursuing that approach, an individually tailored, more aggressive treatment (e.g., more radical resection for *IDH1‐R132G*‐mutated or *IDH2‐R172T*‐mutated CS) and performance of more frequent restaging controls addressing a possibly higher tendency of recurrence could be achieved. However, the statistical significance of these findings from the *IDH1/2* subgroup analysis should be interpreted with caution due to the relatively small number of patients and the limited number of events.

Importantly, *IDH2* mutations were strongly associated with dedifferentiated CS, a subtype known for aggressive behavior and poor prognosis. Although earlier studies have reported high *IDH* mutation rates in high‐grade tumors,^2^, ^16^, ^17^, ^18^, ^19^, ^26^ our data are the first to explicitly demonstrate that this enrichment is specific to *IDH2* rather than *IDH1*. This insight contributes to the growing understanding that *IDH1* and *IDH2* are not functionally interchangeable and may drive distinct biological pathways in tumor progression.

The prognostic impact of *IDH* mutations in CS has been a matter of ongoing controversies. Although some studies reported improved progression‐free survival in *IDH*‐mutated high‐grade CS,^9^ others failed to demonstrate survival differences^27^, ^30^ or even report a negative impact of *IDH* mutation on overall survival.^16^, ^17^, ^19^, ^26^, ^28^, ^29^ In contrast, we observed a marked reduction in DSS among patients with *IDH2* mutations (33% of patients died of disease‐related causes), independent of histological grade. *IDH2*‐mutated patients had a median DSS of 97 months, significantly lower than both *IDH1*‐mutated (163 months) and *IDH*‐WT tumors (210 months). Multivariable Cox regression confirmed *IDH2* mutation as an independent predictor of poor DSS (hazard ratio, 3.61; *p* = .020). In line with our findings, Nicolle et al.^31^ could impressively demonstrate that the combination of *IDH* mutations with high mitotic state and regional 14q32 loss of expression has superior clinical value as compared to the current established grading system. The fact that the driving force of *IDH* mutation status alone seems not strong enough to reach comparable predictability for progression of disease possibly reflects that the combination of different molecular profiles, as well as a dedifferentiated histopathology even more reliably determines the aggressiveness of CS. Interestingly, despite their worse DSS, patients with *IDH2*‐mutant tumors did not show significantly higher rates of local recurrence or metastasis. However, the trend toward shorter MFS and RFS suggests that *IDH2* mutation may contribute to more aggressive disease dynamics over time. This finding is consistent with the observation that *TP53* co‐mutations were only found in dedifferentiated CS. Cross et al.^19^ also found a significantly shorter time interval of only 6 months between diagnosis and detection of first metastatic disease in *IDH2*‐mutant compared to *IDH1*‐mutant or *IDH‐WT* CS patients, lending further support to our hypothesis. The absence of statistical significance in MFS and RFS may be due to the limited number of events and patients with *IDH2* mutations (*n* = 23), warranting further investigation in larger cohorts.

Our analysis also revealed that *IDH2*‐mutated CS occurred predominantly in older patients (mean age 65.6 years), consistent with prior studies.^17^, ^19^, ^31^, ^32^ This age association may reflect the slow growth and late detection of *IDH2*‐mutant tumors until they dedifferentiate and thus rapidly affect the prognosis of the patients. An alternative explanation for this finding is an accumulation of additional molecular alterations over time. Indeed, the literature suggests that telomerase activation via *TERT‐p* mutations, more frequent in older *IDH2*‐mutant patients, may contribute to delayed senescence and increased malignant transformation.^19^, ^33^ Similarly, in a very recent meta analysis encompassing a final cohort of 1152 patients sourced from 21 studies, Swayambunathan et al.^32^ revealed that most patients with *IDH2*‐mutant CS were of an age between 61 and 80 years (44.8%) as opposed to age groups <40 years (13,8%) and 41–60 years (32.8%), suggesting that the tumors are more advanced.

Tumor size is another important prognostic factor. We identified a tumor size larger than 8 cm as a significant negative predictor of DSS, RFS, and MFS. Although *IDH2*‐mutated tumors showed a trend toward larger size, this did not reach statistical significance, possibly due to limited data availability (only 77% had complete volumetric information). Prior studies have associated larger tumor size with *IDH2* mutations and worse outcomes,^19^, ^26^, ^34^ reinforcing our interpretation. Anatomical distribution also differed by *IDH* status. All *IDH2*‐mutated tumors in our cohort were located in the extremities, particularly the lower limbs. No *IDH2* mutations were found in the axial skeleton (spine, pelvis, or chest wall). This pattern is consistent with findings from other authors, who either did not found *IDH*‐mutated CS in the spinal region,^16^ reported no *IDH2*‐mutated CS in the spinal region,^17^ or observed a strong association between *IDH2* mutations and appendicular localization.^19^, ^32^ This distinct distribution may reflect site‐specific microenvironments or developmental pathways that influence mutation acquisition.

At the molecular level, we examined co‐mutations in *TP53*, *TERT‐p*, and *CDKN2A/B*. *TP53* mutations were significantly associated with poor DSS, MFS, and RFS and were more frequently found in *IDH*‐mutated and dedifferentiated tumors. Regarding the DSS, the Cox regression analysis revealed no significant influence of the *TP53* mutation; here, the impact of dedifferentiation and the *IDH2* mutation was predominant. Remarkably, the situation was reversed in MFS and RFS: here, a significant negative influence of the *TP53* mutation was demonstrated, whereas the *IDH2* mutation appears to have no influence. Although *TP53* mutations occurred across *IDH* subgroups, they were more common in *IDH1*‐mutated and *IDH2*‐mutated CS. Nevertheless, these were only detected in dedifferentiated CS, suggesting a potentially cooperative role in tumor progression. This is in line with prior studies showing that *TP53* mutations are enriched in high‐grade CS and predict adverse outcomes,^18^, ^19^, ^35^ reinforcing the perspective that they should be kept in mind as a potentially actionable target that, to date, are neither fully explored nor used.

*TERT‐p* mutations, known to be a causative factor for disease progression and dedifferentiation, have been initially described to play a decisive role also in CS by Martin et al.^36^ In our study, they were present in only 8% of cases and, although more frequent in *IDH*‐mutant tumors (especially *IDH2*), did not reach statistical significance in survival analysis. Because of the small number of *TERT‐p*–mutated cases, our study may be underpowered to detect subtle effects. Other studies have linked *TERT‐p* mutations to dedifferentiation and poor prognosis in CS,^9^, ^18^, ^19^, ^33^ but these findings remain inconsistent.

*CDKN2A/B* deletions were rare in our cohort (5%), with the highest mutation rates in *IDH1*‐mutated and *IDH2*‐mutated (9% and 5%, respectively) CS as opposed to only 2% in *IDH‐WT* CS, and did not show significant associations with survival or *IDH* mutation status. Differences in detection methods (e.g., NGS vs. fluorescence in situ hybridization) may account for variability in reported frequencies across studies.^16^, ^17^, ^18^ Although prior work suggests a role for *CDKN2A/B* loss in dedifferentiation and progression, our data are insufficient to support this conclusively.

One of the most novel aspects of our analysis is the variant‐specific survival data for *IDH1* and *IDH2* mutations. Among *IDH1* variants, *R132C* conferred better DSS than *R132G*. Within *IDH2*, *R172S* had the most favorable prognosis, whereas *R172T* was associated with the poorest DSS and MFS. These findings suggest that the specific amino acid substitution in the *IDH* enzymes has functional relevance for tumor behavior. Although prior studies have not consistently analyzed variant‐level outcomes,^19^, ^28^ our data point to the value of deeper mutational profiling in CS.

The comparatively high number of cases is a particular strength of this study with detailed clinical data, long‐term follow‐up, and a rigorous molecular analysis, which enabled us to clarify the previous controversy regarding the influence of the IDH mutation on patient’s survival.

Despite the strengths, limitations must be acknowledged. First, the relatively small number of *IDH2*‐mutant cases may limit the generalizability of our findings. Second, due to the retrospective nature, not all patients had complete imaging or volumetric data. Finally, the rarity of *TERT‐p* and *CDKN2A/B* alterations in our cohort limits statistical power to assess their impact. Although tumor‐only sequencing methods have limitations, several studies have demonstrated their feasibility and accuracy in term of somatic mutation detection.^21^, ^22^

## CONCLUSION

In conclusion, our study provides, with a sizably large number of cases, the first comprehensive evidence that *IDH2* and *TP53* mutations in CS are enriched in dedifferentiated tumors and—independently of tumor grade—predict poor survival. These tumors are more likely to occur in older patients, involve the extremities, and harbor co‐mutations. Although the role of *TERT‐p* and *CDKN2A/B* remains unclear, our findings emphasize the need for *IDH2*‐focused molecular risk stratification in CS. Unlike *IDH*‐mutant gliomas that additionally have distinct morphology and more regularly co‐occurring molecular alterations, *IDH*‐mutant and *IDH‐WT* CS are histologically identical and are—regardless of their different genetic profile and biological behavior—diagnosed and treated in the same way.^29^ Therefore, future research should explore targeted therapeutic strategies for *IDH2*‐mutant CS (such as novel drugs known to specifically inhibit mutant *IDH1/2*) and investigate the use of 2‐HG as a potential diagnostic or monitoring biomarker. Following that concept holds the key to more precisely match‐selective therapy with underlying pathway mechanisms and to improve individual outcome, particularly for progressive and refractory CS disease.

## AUTHOR CONTRIBUTIONS

**Anne Weidlich**: Conceptualization, methodology, software, data curation, investigation, formal analysis, funding acquisition, visualization, writing–original draft, and writing–review and editing. **Klaus‐Dieter Schaser**: Conceptualization, funding acquisition, investigation, methodology, project administration, resources, supervision, validation, and writing–review and editing. **Tareq A. Juratli**: Conceptualization, funding acquisition, investigation, methodology, supervision, validation, and writing–review and editing. **Jessica Pablik**: Investigation, resources, and writing–review and editing. **Sven Märdian**: Investigation, resources, and writing–review and editing. **Kathrin Hauptmann**: Investigation, resources, and writing–review and editing. **Philipp Schwabe**: Investigation, resources, and writing–review and editing. **Stephan Richter**: Investigation, resources, and writing–review and editing. **Hagen Fritzsche**: Investigation, resources, and writing–review and editing.

## CONFLICT OF INTEREST STATEMENT

Tareq A. Juratli reports consulting fees from Ipsen and Stryker Corporation. Sven Märdian reports consulting fees from DePuy Synthes Products, Inc, KLS‐Martin LP, and University Medicine Rostock. Stephan Richter reports consulting fees from PharmaMar, Deciphera, and Boehringer Ingelheim; payment or honoraria from PharmaMar, Deciphera, and Boehringer Ingelheim; payment for expert testimony from PharmaMar, Deciphera, and Boehringer Ingelheim; support for attending meetings and/or travel from PharmaMar, Deciphera, Boehringer Ingelheim; participation on a data safety monitoring board or advisory board for PharmaMar, Deciphera, and Boehringer Ingelheim; and other financial or nonfinancial interests in PharmaMar, Boehringer Ingelheim, and Deciphera. The other authors declare no conflicts of interest.

## Supporting information

Supplementary Material

## Data Availability

The data that supports the findings of this study are available in the supplementary material of this article.
