# Supplementary material for: Presence of IDH2 and TP53 mutations significantly reduces survival of patients with chondrosarcoma
Source: Cancer. 2026 Mar 29;132(7):e70376. doi: 10.1002/cncr.70376 (PMC13033615; doi:10.1002/cncr.70376)
Supplement: Supplementary file 1 — Supplementary Material [file CNCR-132-e70376-s001.docx]

**Supplementary Data**


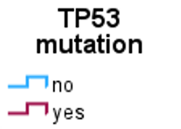

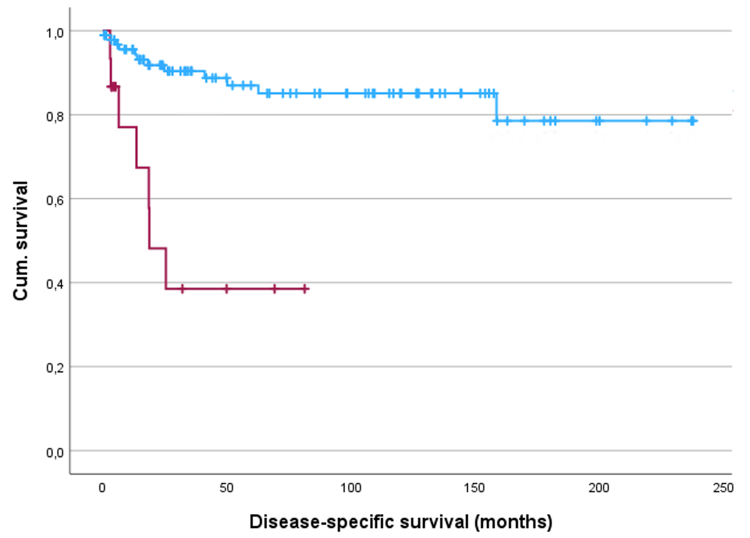


a)


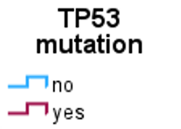

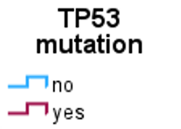

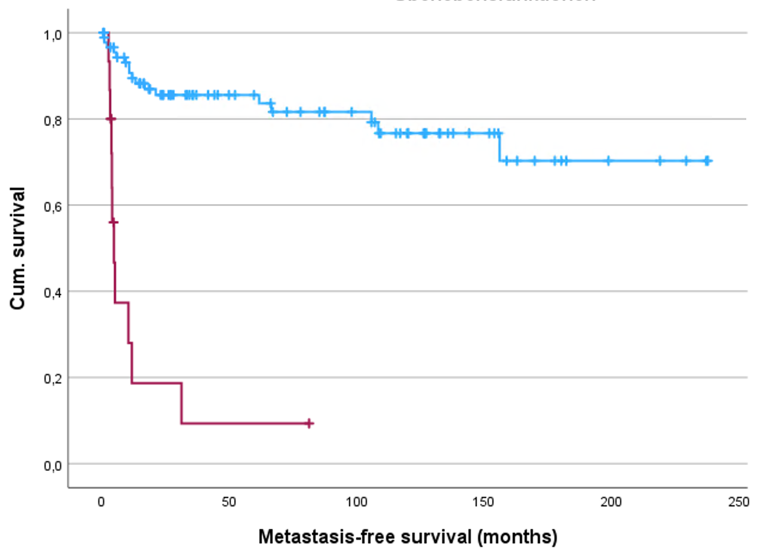

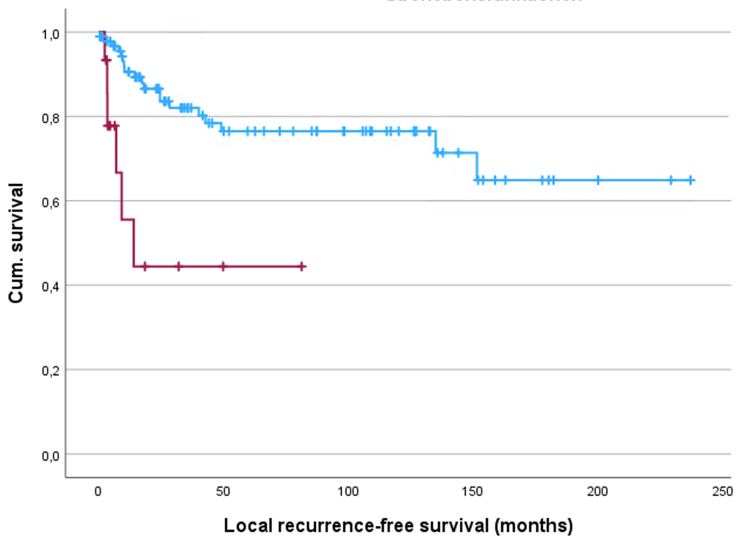


b) c)

**Figure 1:** Significant worse DSS **(a)**, MFS **(b)** and RFS **(c)** with *TP53*-mutation, p<0.001 each.


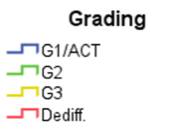

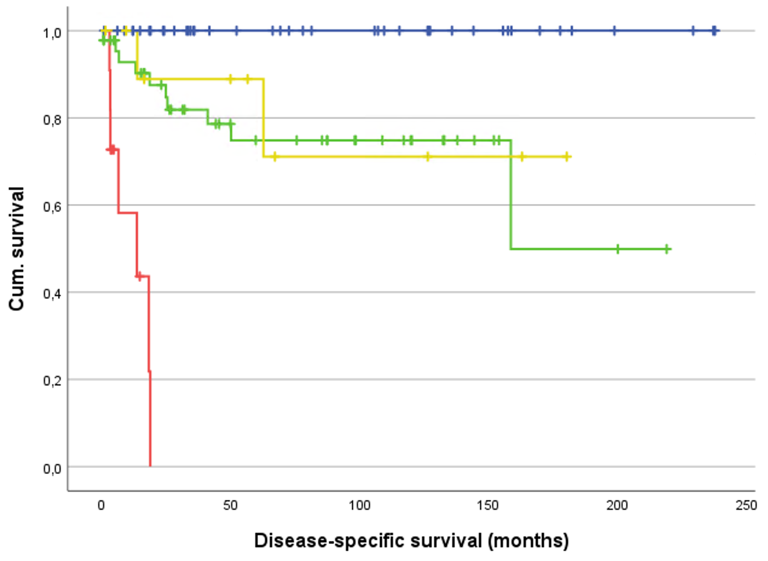


a)


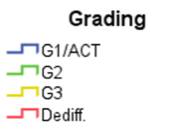

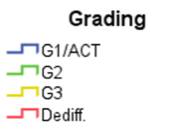

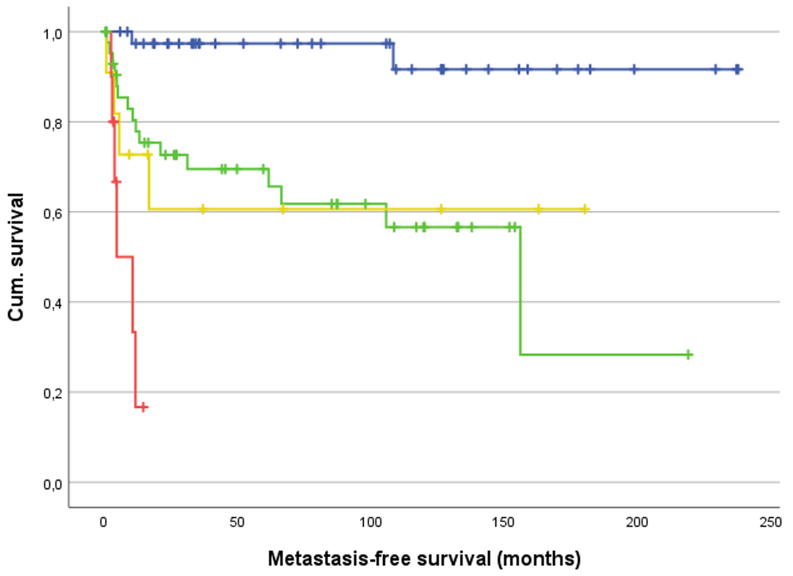

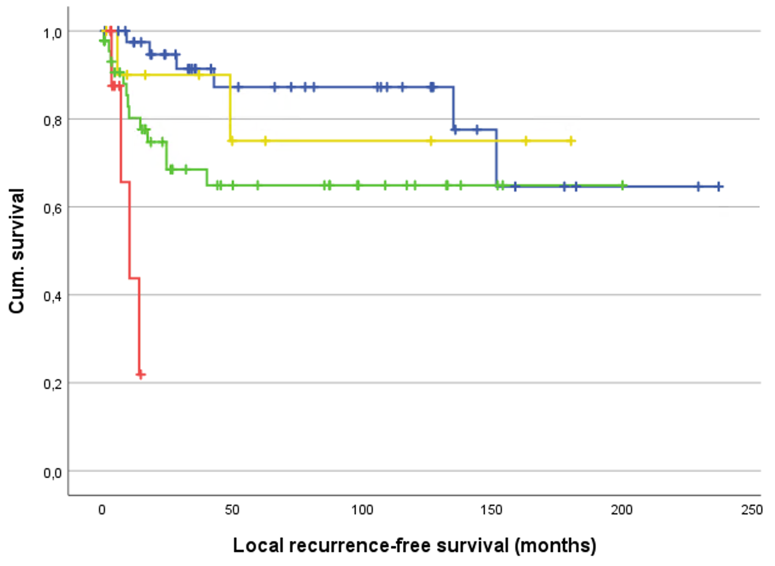


c) d)

**Figure 2:** DSS **(a)**, MFS **(b)** and RFS **(c)** regarding histopathological grading, all significant with p<0.001.
**a):** G1 vs. G2/G3/dediff.: p<0.001/0.003/<0.001, respectively. G2 vs. G3/dediff.: p=0.670/<0.001, respectively. G3 vs. dediff.: p<0.001

**b):** G1 vs. G2/G3/dediff.: p<0.001/0.001/<0.001, respectively. G2 vs. G3/dediff.: p=0.947/<0.001, respectively. G3 vs. dediff: p=0.057

**c):** G1 vs. G2/G3/dediff.: p=0.035/0.733/<0.001, respectively. G2 vs. G3/dediff.: p=0.447/0.033, respectively. G3 vs. dediff.: p=0.017

**Table 1:** Mutation analysis

|  | | **All n = 109 (%)** | **IDH wild type n = 45 (%)** | ***IDH1* mutation n = 41 (%)** | ***IDH2* mutation n = 23 (%)** | **χ²-test p =** |
| --- | --- | --- | --- | --- | --- | --- |
| ***IDH1***  **variant** | **R132C** | 25 | - | 25 (61.0) | - | - |
|  | **R132L** | 3 | - | 3 (7.3) | - |  |
|  | **R132G** | 9 | - | 9 (22.0) | - |  |
|  | **R132H** | 3 | - | 3 (7.3) | - |  |
|  | **R132S** | 1 | - | 1 (2.4) | - |  |
| ***IDH2***  **variant** | **R172S** | 8 | - | - | 8 (34.8) | - |
|  | **R172G** | 5 | - | - | 5 (21.7) |  |
|  | **R172M** | 7 | - | - | 7 (30.4) |  |
|  | **R172T** | 3 | - | - | 3 (13.0) |  |
| ***TP53* mutation** | | 15 (13.8) | 4 (8.9) | 7 (17.1) | 4 (13.8) | 0.464 |
| ***TERT-p* mutation** | | 9 (8.3) | 1 (2.2) | 5 (12.2) | 3 (13.0) | 0.157 |
| ***CDKN2A/B* deletion** | | 5 (4.6) | 1 (2.2) | 2 (4.9) | 2 (8.7) | 0.480 |

**Table 2:** Univariate regression analysis (log rank test)

|  | **Disease-specific survival**  **p =** | **Metastasis-free survival****  **p =** | **Recurrence-free survival**  **p =** |
| --- | --- | --- | --- |
| **Age > 60 years** | **0.018*** | 0.280 | 0.535 |
| **Sex** | 0.438 | 0.193 | 0.974 |
| **Precursor lesion** | 0.327 | 0.526 | 0.368 |
| **Localization:** | 0.709 | 0.443 | 0.587 |
| **Extremities** | 0.669 | 0.218 | 0.302 |
| **Spine** | 0.331 | 0.149 | 0.145 |
| **Pelvis** | 0.272 | 0.378 | 0.552 |
| **Chest wall / Scapula** | 0.428 | 0.563 | 0.615 |
| **Tumor size >8cm** | **0.007*** | **0.039*** | **<0.001*** |
| **R0 resection°** | 0.600 | 0.525 | 0.611 |
| **Grading** | **<0.001*** | **<0.001*** | **<0.001*** |
| **Neoadjuvant chemotherapy** | 0.377 | 0.939 | 0.321 |
| **Adjuvant chemotherapy** | 0.990 | 0.226 | **0.026*** |
| **Neoadjuvant radiation** | 0.580 | 0.534 | 0.493 |
| **Adjuvant radiation** | 0.608 | 0.269 | 0.940 |
| ***IDH-WT* vs. *IDH*mut** | **0.043*** | 0.199 | 0.965 |
| ***IDH-WT* vs. *IDH1* vs. IDH2** | **0.001*** | 0.255 | 0.702 |
| ***IDH1* variant** | **0.037*** | 0.711 | 0.167 |
| ***IDH2* variant** | **<0.001*** | **0.002*** | 0.937 |
| ***TP53* mutation** | **<0.001*** | **<0.001*** | **<0.001*** |
| ***TERT-p* mutation** | 0.689 | 0.605 | 0.323 |
| ***CDKN2A/B* deletions** | 0.363 | 0.856 | 0.272 |
| **Local recurrence** | **0.005*** | **<0.001*** | - |
| **Metastases** | **<0.001*** | - | **<0.001*** |

* Significant in 95% confidence interval

** without 2 patients with primary metastases, n=107

° without intralesional planned resections, n=84
